# On the evolutionary *p*-Laplacian equation with a partial boundary value condition

**DOI:** 10.1186/s13660-018-1820-x

**Published:** 2018-08-31

**Authors:** Huashui Zhan

**Affiliations:** School of Applied Mathematics, Xiamen University of Technology, Xiamen, China

**Keywords:** 35L65, 35L85, 35R35, Nonlinearity, Stability, *p*-Laplacian equation, Partial boundary value condition

## Abstract

Consider the equation

$${u_{t}} = \operatorname{div} \bigl(d^{\alpha} \vert \nabla u \vert ^{p - 2}\nabla u\bigr) + \frac{\partial b_{i}(u,x,t)}{\partial{x_{i}}},\quad (x,t) \in\Omega \times(0,T), $$

where Ω is a bounded domain, $d(x)$ is the distance function from the boundary *∂*Ω. Since the nonlinearity, the boundary value condition cannot be portrayed by the Fichera function. If $\alpha< p-1$, a partial boundary value condition is portrayed by a new way, the stability of the weak solutions is proved by this partial boundary value condition. If $\alpha>p-1$, the stability of the weak solutions may be proved independent of the boundary value condition.

## Introduction and the main results

Benedikt et al. [1] considered the equation

1.1 $$ {u_{t}} = \operatorname{div} \bigl({ \vert {\nabla u} \vert ^{p - 2}} \nabla u\bigr) + q(x) \vert u \vert ^{\gamma-1}u , \quad(x,t) \in{Q_{T}}=\Omega \times(0,T) , $$

and showed that the uniqueness of the solution is not true [1]. Here, $0<\gamma<1$, Ω is a bounded domain in $R^{N}$ with appropriately smooth boundary, $q(x)\geq0$ and at least there is a $x_{0}\in\Omega$ such that $q(x_{0})>0$. Zhan [2] had shown that the stability of the solutions to the equation

1.2 $$ {u_{t}} = \operatorname{div}\bigl(d^{\alpha} \vert \nabla u \vert ^{p - 2} \nabla u\bigr)+f(u,x,t), \quad (x,t) \in{Q_{T}}, $$

is true, where $d(x) = \operatorname{dist} (x,\partial\Omega)$ is distance function, $\alpha>0$ is a constant. The result of [2] is in complete antithesis to that of [1]. So, when the well-posedness of the solutions is considered, the degeneracy of the diffusion coefficient $d^{\alpha}$ plays an important role.

Yin and Wang [3, 4] studied the equation

1.3 $$ {u_{t}} = \operatorname{div} \bigl(d^{\alpha}{ \vert {\nabla u} \vert ^{p - 2}}\nabla u\bigr),\quad(x,t) \in{Q_{T}}, $$

and showed that there is a constant $\gamma>1$ such that, if $\alpha< p-1$, then

1.4 $$ \iint_{Q_{T}} \vert \nabla u \vert ^{\gamma}\,dx\,dt< \infty. $$

Recently, Zhan [5] had generalized the Yin and Wang result to the equation

1.5 $$ {u_{t}} = \operatorname{div} \bigl(d^{\alpha} \vert \nabla u \vert ^{p - 2}\nabla u\bigr) + \sum_{i=1}^{N} \frac{\partial b_{i}(u)}{\partial{x_{i}}},\quad(x,t) \in{Q_{T}}. $$

In this paper, we continue to consider a more general equation,

1.6 $$ {u_{t}} = \operatorname{div} \bigl(d^{\alpha} \vert \nabla u \vert ^{p - 2}\nabla u\bigr) + \sum_{i=1}^{N} \frac{\partial b_{i}(u,x,t)}{\partial {x_{i}}},\quad(x,t) \in{Q_{T},} $$

and study the well-posedness of the weak solutions. As usual, the initial value

1.7 $$ u(x,0)=u_{0}(x), \quad x\in\Omega, $$

is necessary. But, since the coefficient $d^{\alpha}$ is degenerate on the boundary, when $\alpha< p-1$, though (1.4) is true, and the boundary value condition

1.8 $$ u(x,t)=0, \quad (x,t)\in\partial\Omega\times(0,T), $$

can be imposed in the sense of the trace, it may be overdetermined. While $\alpha\geq p-1$, it is almost impossible to prove (1.4). How to impose a suitable boundary value condition to match up with Eq. (1.6) becomes very troublesome [4]. Stated succinctly, instead of the Dirichlet boundary value condition (1.8), only a partial boundary value condition,

1.9 $$ u(x,t)=0, \quad (x,t)\in\Sigma_{p}\times(0,T), $$

is needed, where $\Sigma_{p}\subseteq\partial\Omega$ is a relatively open subset. The main difficulty comes from the fact that, since Eq. (1.6) is a nonlinear parabolic equation, $\Sigma_{p}$ cannot be expressed by the Fichera function (one can refer to Sect. 6 of this paper). In this paper, we will try to depict the geometric characteristic of $\Sigma_{1}$, and establish the stability of the weak solutions based on the partial boundary value condition (1.9).

We denote

$$W^{1,p}_{\alpha}=\biggl\{ u\in W^{1, p}_{\mathrm {loc}}( \Omega): \int_{\Omega}d^{ \alpha} \vert \nabla u \vert ^{p}\,dx< \infty\biggr\} . $$

### Definition 1.1

Let

1.10 $$ u \in L^{\infty}(Q_{T}), \quad\quad u_{t}\in L^{2}(Q_{T}), \quad \quad {d^{\alpha}} { \vert {\nabla u} \vert ^{p}} \in{L^{\infty}}\bigl(0,T;L^{1}(\Omega ) \bigr), $$

and

1.11 $$ \iint_{{Q_{T}}} \Biggl[ u_{t}(\varphi_{1} \varphi_{2}) + d^{\alpha} \vert \nabla u \vert ^{p- 2} \nabla u \cdot\nabla(\varphi_{1} \varphi_{2}) + \sum _{i=1}^{N}b_{i}(u,x,t) ( \varphi_{1}\varphi_{2})_{x _{i}} \Biggr] \,dx\,dt= 0. $$

Here $\varphi_{1} \in{ C_{0}^{1} ({Q_{T}})}$, $\varphi_{2}(x,t) \in W^{1,p}_{\alpha}$ for any given *t*, and $\vert \varphi_{2}(x,t) \vert \leq c$ for any given *x*. If the initial value (1.7) is satisfied in the sense of

1.12 $$ \lim_{t\rightarrow0} \int_{\Omega} \bigl\vert u(x,t) - u_{0}(x) \bigr\vert \,dx = 0, $$

then we say $u(x,t)$ is a solution of Eq. (1.6) with the initial condition (1.7).

### Theorem 1.2

*If* $p>2$ *and* $\alpha<\frac{p-2}{2}$, *for any* $i \in\{ 1,2,\ldots, N\} $, $b_{i}(s,x,t)$ *is a* $C^{1}$ *function*, *and there are constants* *β*, *c* *such that*

1.13 $$\begin{aligned}& \bigl\vert {{b_{i}}(s,x,t)} \bigr\vert \leqslant c{ \vert s \vert ^{1 + \beta}},\quad\quad \biggl\vert \frac{\partial b_{i}(s,x,t)}{\partial s} \biggr\vert \leqslant c{ \vert s \vert ^{\beta}},\quad\quad \biggl\vert \frac{b_{i}(s,x,t)}{ \partial x_{i}} \biggr\vert \leqslant c, \\& \quad i=1,2,\ldots, N, \end{aligned}$$

1.14 $$\begin{aligned}& {u_{0}} \in{L^{\infty}}(\Omega)\cap W^{1,p}(\Omega), \end{aligned}$$

*then there is a solution of Eq*. (1.6) *with the initial value* (1.7).

Certainly, we suggest that the conditions in Theorem 1.2 are not the optimal, we only provide a basic result of the existence here. The main aim of this paper is to research the stability of the weak solutions.

### Theorem 1.3

*Let* $\alpha>p-1>0$, $b_{i}$ *satisfy*

1.15 $$ \bigl\vert b_{i}(u,x,t)-b_{i}(v,x,t) \bigr\vert \leq cd^{\frac{\alpha}{p}} \vert u-v \vert , \quad i=1,2,\ldots, N. $$

*If* *u* *and* *v* *are two solutions of Eq*. (1.6) *with the initial values* $u_{0}(x)$ *and* $v_{0}(x)$, *respectively*, *then*

1.16 $$ \int_{\Omega} \bigl\vert u(x,t) - v(x,t) \bigr\vert \,dx \leqslant c \int_{\Omega} \bigl\vert u_{0}(x)- v_{0}(x) \bigr\vert ,\quad\forall t \in[0,T). $$

### Remark 1.4

If $\alpha< p-1$, we can prove the stability of the weak solutions for the initial-boundary value problem (1.6), (1.7), and (1.8) in a standard way [6]. We ask whether the spatial variable *x* in the nonlinear convection term $b_{i}(u,x,t)$ can bring about the essential change. In particular, when $b_{i}(s,x,t)\equiv0$, then only if $\alpha\geq p-1$, Yin and Wang [3] had shown that

$$\int_{\Omega} \bigl\vert u(x,t)-v(x,t) \bigr\vert ^{2}\,dx\leq \int_{\Omega} \bigl\vert u_{0}(x)-v_{0}(x) \bigr\vert ^{2}\,dx. $$

Without the condition (1.15), we can prove a result of the local stability of the weak solutions. This is the following theorem.

### Theorem 1.5

*Let* $p>1$, $b_{i}(s,x,t)$ *be a Lipschitz function*. *If* *u* *and* *v* *are two solutions of Eq*. (1.6), *then there exists a constant* *β* *large enough such that*

1.17 $$ \int_{\Omega}d^{\beta} \bigl\vert u(x,t)-v(x,t) \bigr\vert ^{2}\,dx\leq \int_{\Omega}d^{ \beta} \bigl\vert u_{0}(x)-v_{0}(x) \bigr\vert ^{2}\,dx. $$

Theorem 1.5 implies that the uniqueness of the weak solutions is true only if $\alpha>0$. When $b_{i}(u,x,t)=b_{i}(x)D_{i}u$, i.e., the convection term is just linear, Theorem 1.5 had been proved in paper [7]. When $b_{i}(u,x,t)=b_{i}(u)$, Theorem 1.5 had been proved in [8] very recently. For the sake of simplicity, we will not give the details of the proof of Theorem 1.5 in this paper.

Once more, by introducing a new kind of the weak solutions, choosing a suitable test function, we can prove the following theorems.

### Theorem 1.6

*Let* $\alpha>p-1$, $p>2$, $b_{i}$ *satisfying*

1.18 $$ \bigl\vert b_{i}(u,x,t)-b_{i}(v,x,t) \bigr\vert \leq cd(x) \vert u-v \vert , \quad i=1,2,\ldots, N. $$

*If* *u* *and* *v* *are two solutions of Eq*. (1.6) *with the initial values* $u_{0}(x)$ *and* $v_{0}(x)$, *respectively*, *then*

1.19 $$ \int_{\Omega} \bigl\vert u(x,t) - v(x,t) \bigr\vert \,dx \leqslant \int_{\Omega} \bigl\vert u_{0}(x)- v_{0}(x) \bigr\vert \,dx,\quad\forall t \in[0,T). $$

Theorem 1.6 seems just a minor version of Theorem 1.3. However, on the right hand side of (1.19), there is no constant *c* as in (1.16).

Last but no the least, we will prove the stability of the solutions based on a partial boundary value condition.

### Theorem 1.7

*Let* $b(s,x,t)$ *be a Lipschitz function*, *u* *and* *v* *be two weak solutions of Eq*. (1.6) *with the same partial homogeneous boundary value*

1.20 $$ u| _{\Sigma_{p}\times(0,T)}=0= v| _{\Sigma_{p}\times(0,T)}. $$

*If*

1.21 $$ p>3, \quad\quad p-1>\alpha\geq\frac{p-1}{p-2}, $$

*and there is nonnegative function* $a_{i}(x)$ *such that*

1.22 $$ \bigl\vert b_{i}(u,v,t)-b_{i}(v,x,t) \bigr\vert \leq a_{i}(x) \vert u-v \vert , $$

*then*

1.23 $$ \int_{\Omega} \bigl\vert u(x,t) - v(x,t) \bigr\vert \,dx \leq \int_{\Omega} \bigl\vert u_{0}(x) - v_{0}(x) \bigr\vert \,dx,\quad\forall t \in[0,T). $$

*Here*,

1.24 $$ \Sigma_{p}=\Biggl\{ x\in\partial\Omega: \sum _{i=1}^{N}a_{i}(x)\neq0\Biggr\} . $$

The paper is arranged as follows. In Sect. 1, we have given the basic definition and introduced the main results. In Sect. 2, we prove the existence of the solution to Eq. (1.6) with initial value (1.7). In Sect. 3, we prove Theorem 1.3. In Sect. 4, we give another kind of the weak solutions. By this new definition, we can prove Theorem 1.6. In Sect. 5, we will prove Theorem 1.7. In Sect. 7, we will give an explanation of the reasonableness of the partial boundary value condition.

## The proof of existence

Consider the regularized equation

2.1 $$ u_{t} = \operatorname{div}\bigl(\bigl(d^{\alpha}+\varepsilon \bigr) \vert \nabla u \vert ^{p-2}\nabla u\bigr)+ \sum _{i=1}^{N} \frac{\partial b_{i}(u,x,t)}{\partial{x_{i}}}, \quad (x,t) \in Q_{T}, $$

with the initial boundary conditions

2.2 $$\begin{aligned}& u(x,0) = u_{0\varepsilon}(x), \quad x\in\Omega, \end{aligned}$$

2.3 $$\begin{aligned}& u(x,t) = 0, \quad (x,t)\in\partial\Omega\times(0,T). \end{aligned}$$

Here, $u_{0\varepsilon} \in C^{\infty}_{0}(\Omega)$ and $u_{0 \varepsilon}$ converges to $u_{0}$ in $W_{0}^{1,p}(\Omega)$.

### Proof of Theorem 1.2

Similar to [9], we can easily prove that there exists a weak solution ${u_{\varepsilon}}\in L^{\infty}(0,T;W ^{1,p}_{0}(\Omega))$ of the initial-boundary value problem (2.1)–(2.3),

2.4 $$ \Vert {u_{\varepsilon}} \Vert _{L^{\infty}({Q_{T}})} \leqslant c. $$

Multiplying (2.1) by $u_{\varepsilon}$ and integrating it over $Q_{T}$, by the fact

$$\begin{aligned} & \iint_{Q_{T}} u_{\varepsilon}\frac{\partial b_{i}(u_{\varepsilon},x,t)}{ \partial x_{i}} \,dx\,dt \\ &\quad = - \iint_{Q_{T}} \frac{\partial u_{\varepsilon }}{\partial x_{i}}b_{i}(u_{\varepsilon},x,t) \,dx\,dt \\ &\quad = - \iint_{Q_{T}}\frac{\partial}{\partial x_{i}} \int _{0}^{u_{\varepsilon }} b_{i}(s,x,t)\,ds\,dx\,dt + \iint_{Q_{T}} \int_{0}^{u_{\varepsilon}} b_{ix _{i}}(s,x,t)\,ds\,dx\,dt \\ &\quad = \iint_{Q_{T}} \int_{0}^{u_{\varepsilon}} b_{ix_{i}}(s,x,t)\,ds\,dx\,dt \\ &\quad =0, \end{aligned}$$

we are able to deduce that

2.5 $$ \iint_{{Q_{T}}}d^{\alpha} \vert \nabla u_{\varepsilon} \vert ^{p}\,dx\,dt\leq \iint_{{Q_{T}}}\bigl(d^{\alpha}+ \varepsilon\bigr) \vert \nabla u_{\varepsilon} \vert ^{p}\,dx\,dt \leq c. $$

Then

2.6 $$ \int_{0}^{T} \int_{\Omega_{\lambda}} \vert \nabla u_{\varepsilon} \vert ^{p}\,dx\,dt \leq c(\lambda) $$

for any $\Omega_{\lambda}=\{x\in\Omega, d(x,\partial\Omega)> \lambda\}\subseteq\Omega$, *λ* being a small constant.

Multiplying (2.5) by $u_{\varepsilon t}$, integrating it over $Q_{T}$, then it yields

2.7 $$ \begin{aligned}[b] \iint_{Q_{T}} (u_{\varepsilon t})^{2}\,dx \,dt&= \iint_{{Q_{T}}} \operatorname{div} \bigl(\bigl(d^{\alpha}+ \varepsilon\bigr){{ \vert {\nabla{u_{\varepsilon}}} \vert }^{p - 2}} \nabla{u_{\varepsilon}}\bigr) \cdot{u_{\varepsilon t}}\,dx\,dt \\ &\quad{} +\sum_{i=1}^{N} \iint_{{Q_{T}}}u_{\varepsilon t}\frac{\partial b_{i}(u_{\varepsilon},x,t)}{ \partial x_{i}}\,dx\,dt . \end{aligned} $$

Notice that

$$\vert \nabla u_{\varepsilon} \vert ^{p-2}\nabla u_{\varepsilon} \cdot\nabla u _{\varepsilon t} = \frac{1}{2}\frac{d}{dt} \int_{0}^{ \vert \nabla u _{\varepsilon} \vert ^{2}} s^{\frac{p - 2}{2}}\,ds . $$

Thus,

2.8 $$\begin{aligned}& \iint_{Q_{T}} \operatorname{div} \bigl( \bigl(d^{\alpha}+ \varepsilon\bigr) \vert \nabla u_{\varepsilon} \vert ^{p-2}\nabla u_{\varepsilon}\bigr) \cdot u_{\varepsilon t}\,dx\,dt \\& \quad = - \iint_{Q_{T}} \bigl(d^{\alpha}+\varepsilon\bigr) \vert \nabla u_{\varepsilon} \vert ^{p-2} \nabla u_{\varepsilon}\nabla u_{\varepsilon t}\,dx\,dt \\& \quad = -\frac{1}{2} \iint_{Q_{T}} \bigl(d^{\alpha}+\varepsilon\bigr) \frac{d}{dt} \int_{0}^{ \vert \nabla u_{\varepsilon} \vert ^{2}} s^{ \frac{p - 2}{2}}\,ds \,dx \,dt. \end{aligned}$$

By condition (1.13),

2.9 $$\begin{aligned}& \iint_{Q_{T}} u_{\varepsilon t} \frac{\partial}{\partial x_{i}} b _{i}(u_{\varepsilon},x,t) \,dx\,dt \\& \quad \leqslant \iint_{Q_{T}} \biggl\vert \frac{b _{i}(u_{\varepsilon},x,t)}{\partial u} \biggr\vert \vert u_{\varepsilon x_{i}} \vert \vert u_{\varepsilon t} \vert \,dx\,dt + \iint_{Q_{T}} \biggl\vert \frac{ b_{i}(u_{\varepsilon}, x,t)}{\partial x_{i}} \biggr\vert \vert u_{\varepsilon x_{i}} \vert \vert u_{\varepsilon t} \vert \,dx\,dt \\& \quad \leqslant\frac{1}{4} \iint_{Q_{T}} (u_{\varepsilon t})^{2}\,dx\,dt + c \iint_{Q_{T}} \vert u_{\varepsilon} \vert ^{2\beta} \vert \nabla u _{\varepsilon} \vert ^{2}\,dx\,dt \\& \quad \quad {} +\frac{1}{4} \iint_{Q_{T}} (u_{\varepsilon t})^{2}\,dx\,dt + c \iint_{Q_{T}} \vert \nabla u_{\varepsilon} \vert ^{2}\,dx\,dt. \end{aligned}$$

By Hölder’s inequality and $\alpha\leq\frac{p-2}{2}$,

2.10 $$\begin{aligned}& \iint_{Q_{T}} \vert u_{\varepsilon} \vert ^{2\beta} \vert \nabla u _{\varepsilon} \vert ^{2}\,dx\,dt \\& \quad \leqslant c \iint_{Q_{T}} \vert \nabla u_{\varepsilon} \vert ^{2}\,dx\,dt \\& \quad = c \iint_{Q_{T}} d^{-\frac{2\alpha}{p}} \cdot d^{\frac{2\alpha}{p}} \vert \nabla u_{\varepsilon} \vert ^{2}\,dx\,dt \\& \quad \leq c \biggl( \iint_{Q_{T}} d^{-\frac{2\alpha}{p- 2}}\,dx\,dt \biggr) ^{\frac{p- 2}{p^{-}}} \cdot \biggl( \iint_{Q_{T}} d^{a} \vert \nabla u_{\varepsilon} \vert ^{p}\,dx\,dt \biggr) ^{\frac{2}{p}} \\& \quad \leq c. \end{aligned}$$

Combining (2.7)–(2.10), we have

$$\iint_{{Q_{T}}} (u_{\varepsilon t})^{2}\,dx\,dt + \iint_{{Q_{T}}} \bigl(d^{ \alpha}+\varepsilon\bigr) \frac{d}{dt} \int_{0}^{ \vert \nabla u_{\varepsilon } \vert ^{2}} s^{\frac{p - 2}{2}}\,ds \,dx\,dt \leqslant c, $$

by the inequality, we have

2.11 $$ \iint_{{Q_{T}}} (u_{\varepsilon t})^{2}\,dx\,dt \leqslant c + c \int_{ \Omega}\bigl(d^{\alpha}+\varepsilon\bigr) \vert \nabla u_{0\varepsilon} \vert ^{p}\,dx \leqslant c. $$

Hence, by (2.4), (2.6), (2.11), there exist a function *u* and a *n*-dimensional vector $\overrightarrow{\zeta}= ({\zeta_{1}},\ldots,{\zeta_{n}})$ satisfying

2.12 $$ u \in{L^{\infty}}({Q_{T}}),\quad \quad\frac{\partial u}{\partial t} \in{L^{2}}({Q_{T}}),\quad\quad \vert {\overrightarrow{ \zeta}} \vert \in L^{1}\bigl(0,T; L^{\frac{p}{p- 1}}(\Omega) \bigr), $$

and $u_{\varepsilon}\rightarrow u$ a.e. $\in Q_{T}$,

$$\begin{aligned}& {u_{\varepsilon}}\rightharpoonup u, \quad \text{weakly star in } L^{\infty }(Q_{T}), \\& u_{\varepsilon} \to u,\quad \text{in }L^{2}\bigl(0,T;L_{\mathrm {loc}}^{r}( \Omega)\bigr), \\& \frac{\partial u_{\varepsilon}}{\partial t} \rightharpoonup\frac{ \partial u}{\partial t} \quad \text{in } {L^{2}}({Q_{T}}), \\& d^{\alpha} \vert \nabla u_{\varepsilon} \vert ^{p- 2} \nabla{u_{ \varepsilon}} \rightharpoonup\overrightarrow{\zeta}\quad \text{in } {L^{1}\bigl(0,T; L^{\frac{p}{{p- 1}}}}(\Omega)\bigr). \end{aligned}$$

Here, if $p\geq2$, $r=2$, while $1< p<2$, $1< r<\frac{Np}{N-p}$.

In order to prove that *u* is the solution of Eq. (1.6), for any function $\varphi\in C_{0}^{1} ({Q_{T}})$, we have

$$\iint_{Q_{T}} \Biggl[ u_{\varepsilon t}\varphi+ \bigl(d^{\alpha}+\varepsilon \bigr) \vert \nabla u_{\varepsilon} \vert ^{p - 2}\nabla u_{\varepsilon} \cdot\nabla\varphi+\sum _{i=1}^{N} b_{i}(u_{\varepsilon},x,t) \varphi_{x_{i}} \Biggr] \,dx\,dt =0, $$

we let $\varepsilon\rightarrow0$.

Since as $\varepsilon\rightarrow0$, by $d(x)>0$, $x\in\Omega$, then $c>\sup_{\operatorname{supp} \varphi}\frac{ \vert \nabla\varphi \vert }{d^{\alpha}}>0$ due to $\varphi\in C_{0}^{1} ({Q_{T}})$, we have

$$\begin{aligned}& \varepsilon \biggl\vert \iint_{Q_{T}} \vert \nabla u_{\varepsilon} \vert ^{p-2}\nabla u_{ \varepsilon}\cdot\nabla\varphi \,dx\,dt \biggr\vert \\& \quad \leq\varepsilon\sup_{\operatorname{supp} \varphi}\frac{ \vert \nabla\varphi \vert }{d^{ \alpha}} \iint_{Q_{T}}\bigl( \vert \nabla u_{\varepsilon} \vert ^{p}+c\bigr)\,dx\,dt\rightarrow 0. \end{aligned}$$

By this note, we have

$$\begin{aligned}& \iint_{Q_{T}}\vec{\zeta}\cdot\nabla\varphi \,dx\,dt \\& \quad = \lim_{\varepsilon\rightarrow0} \iint_{Q_{T}}d^{\alpha} \vert \nabla u _{\varepsilon} \vert ^{p-2}\nabla u_{\varepsilon}\cdot\nabla\varphi \,dx\,dt \\& \quad = \lim_{\varepsilon\rightarrow0} \iint_{Q_{T}}\bigl(d^{\alpha}+\varepsilon \bigr) \vert \nabla u_{\varepsilon} \vert ^{p-2}\nabla u_{\varepsilon}\cdot\nabla \varphi \,dx\,dt -\lim_{\varepsilon\rightarrow0}\varepsilon \iint_{Q _{T}} \vert \nabla u_{\varepsilon} \vert ^{p-2}\nabla u_{\varepsilon}\cdot \nabla\varphi \,dx\,dt \\& \quad = \lim_{\varepsilon\rightarrow0} \iint_{Q_{T}}\bigl(d^{\alpha}+\varepsilon \bigr) \vert \nabla u_{\varepsilon} \vert ^{p-2}\nabla u_{\varepsilon}\cdot\nabla \varphi \,dx\,dt. \end{aligned}$$

Now, similar to the general evolutionary *p*-Laplician equation [6], we are able to prove that (the details are omitted here)

2.13 $$ \iint_{{Q_{T}}} \bigl[ u \varphi_{t}+ \vec{\varsigma} \cdot\nabla \varphi+b_{i}(u,x,t)\varphi_{x_{i}} \bigr] \,dx \,dt= 0 $$

and

2.14 $$ \iint_{{Q_{T}}} d^{\alpha} \vert {\nabla u} \vert ^{p - 2}\nabla u \cdot\nabla\varphi \,dx\,dt = \iint_{{Q_{T}}} \overrightarrow{\zeta} \cdot\nabla\varphi \,dx \,dt, $$

for any function $\varphi\in C_{0}^{1} ({Q_{T}})$. Then

2.15 $$ \iint_{{Q_{T}}} \Biggl[ u_{t}\varphi+ d^{\alpha} \vert \nabla u \vert ^{p- 2}\nabla u \cdot\nabla\varphi+\sum _{i=1}^{N}b_{i}(u,x,t) \varphi_{x_{i}} \Biggr] \,dx\,dt = 0. $$

If for any given $t\in[0, T)$, we denote $\Omega_{\varphi}=\operatorname{supp} \varphi$, then

2.16 $$ \int_{0}^{T} \int_{\Omega_{\varphi}} \Biggl[ u_{t}\varphi+ d^{\alpha} \vert \nabla u \vert ^{p- 2}\nabla u \cdot\nabla\varphi+\sum _{i=1} ^{N}b_{i}(u,x,t) \varphi_{x_{i}} \Biggr] \,dx\,dt = 0. $$

Now, for any $\varphi_{1}\in C_{0}^{1} ({Q_{T}})$, $\varphi_{2}(x,t) \in W^{1,p}_{\alpha}$ for any given *t*, and $\vert \varphi_{2}(x,t) \vert \leq c$ for any given *x*, it is clear that $\varphi_{2}\in W^{1, p}( \Omega_{\varphi_{1}})$. By the fact that $C_{0}^{\infty}( \Omega_{\varphi_{1}})$ is dense in $W^{1, p}(\Omega_{\varphi_{1}})$, by a process of limits, we have

2.17 $$ \begin{aligned}[b] & \int_{0}^{T} \int_{\Omega_{\varphi_{1}}} \Biggl[ u_{t}(\varphi_{1} \varphi _{2}) + d^{\alpha} \vert \nabla u \vert ^{p- 2}\nabla u \cdot \nabla(\varphi_{1}\varphi_{2}) \\&\quad{} +\sum_{i=1}^{N}b_{i}(u,x,t) ( \varphi _{1}\varphi_{2})_{x_{i}} \Biggr] \,dx\,dt = 0,\end{aligned} $$

which implies that

2.18 $$ \int_{0}^{T} \int_{\Omega} \bigl[ u_{t}(\varphi_{1} \varphi_{2}) + d ^{\alpha} \vert \nabla u \vert ^{p- 2}\nabla u \cdot\nabla(\varphi _{1}\varphi_{2})+b_{i}(u,x,t) (\varphi_{1}\varphi_{2})_{x_{i}} \bigr] \,dx\,dt = 0. $$

Then *u* satisfies Eq. (1.6) in the sense of Definition 1.1. □

## Proof of Theorem 1.3

### Proof

Let *u* and *v* be two weak solutions of Eq. (1.6) with the initial values $u_{0}(x)$ and $v_{0}(x)$, respectively. For large enough $n>0$, let

3.1 $$ g_{n}(s)= \int_{0}^{s}h_{n}(\tau)\,d\tau, h_{n}(s)=2n\bigl(1- \vert ns \vert \bigr)_{+}. $$

Obviously $h_{n}(s)\in C(\mathbb{R})$, and

3.2 $$ \begin{gathered} h_{n}(s)\geq0,\quad\quad \bigl\vert sh_{n}(s) \bigr\vert \leq1, \quad\quad \bigl\vert g_{n }(s) \bigr\vert \leq1;\\ \lim_{n \rightarrow\infty}g_{n }(s)=\operatorname{sign}s, \quad\quad \lim_{n \rightarrow\infty}sh_{n}(s)=0.\end{gathered} $$

We define

$${d_{n}}(x) = \textstyle\begin{cases} nd(x),&d(x)< \frac{1}{n}, \\ 1,&d(x)\geq\frac{1}{n}. \end{cases} $$

Since for any given *t*, $\varphi_{1}=g_{n}(u-v)\in W^{1,p}_{\alpha}$, by a process of limit, we can choose $d_{n}{g_{n}}(u - v)$ as the test function, then

3.3 $$\begin{aligned}& \int_{{\Omega}} d_{n}(x)g_{n}(u - v) \frac{\partial(u - v)}{\partial t}\,dx \\& \quad {} + \int_{{\Omega}} d^{\alpha}\bigl( \vert {\nabla u} \vert ^{p- 2}\nabla u - \vert {\nabla v} \vert ^{p - 2}\nabla v\bigr) \cdot\nabla(u - v)h _{n}(u-v)d_{n}(x)\,dx \\& \quad {} + \int_{{\Omega}} d^{\alpha}\bigl( \vert {\nabla u} \vert ^{p- 2}\nabla u - \vert {\nabla v} \vert ^{p- 2}\nabla v \bigr) (u - v){g}_{n}(u-v) \nabla d_{n}\,dx \\& \quad {} + \int_{{\Omega}} \bigl({b_{i}}(u,x,t) - {b_{i}}(v,x,t)\bigr) \cdot(u - v)_{x _{i}}{h}_{n}(u-v)d_{n}(x) \,dx \\& \quad {} + \int_{{\Omega}} \bigl({b_{i}}(u,x,t) - {b_{i}}(v,x,t)\bigr) \cdot{g}_{n}(u-v)d _{nx_{i}}(x)\,dx = 0. \end{aligned}$$

Thus

3.4 $$\begin{aligned}& \lim_{n\rightarrow\infty} \int_{\Omega} d_{n}(x){g_{n}}(u - v)\frac{ \partial(u - v)}{\partial t}\,dx = \frac{d}{dt} \Vert {u - v} \Vert _{L^{1}(\Omega)}, \end{aligned}$$

3.5 $$\begin{aligned}& \int_{\Omega} d^{\alpha}\bigl( \vert \nabla u \vert ^{p - 2}\nabla u - \vert \nabla v \vert ^{p- 2}\nabla v\bigr) \cdot\nabla(u - v)h{_{n}}(u-v)d _{n}(x)\,dx\geq0. \end{aligned}$$

Denoting $D_{n}=\{x\in\Omega:d(x)>\frac{1}{n}\}$, $q=\frac{p}{p-1}$, clearly

$$\begin{aligned} \bigl\Vert nd^{\frac{\alpha}{p}} \bigr\Vert _{L^{p}(\Omega\setminus D_{n})}&=n \bigl\Vert d^{\frac{ \alpha}{p}} \bigr\Vert _{L^{p}(\Omega\setminus D_{n})} \\ &=n \biggl( \int_{\Omega\setminus D_{n}}d^{\alpha}\,dx \biggr) ^{ \frac{1}{p}}\leq cn^{1-\frac{1+\alpha}{p}}, \end{aligned}$$

which goes to zero since that $\alpha>p-1$.

By this fact, $\vert \nabla d_{n} \vert =n$, $x\in{\Omega\setminus D_{n}}$, we have

3.6 $$\begin{aligned}& \biggl\vert \int_{\Omega} d^{\alpha}\bigl( \vert \nabla u \vert ^{p- 2}\nabla u - \vert \nabla v \vert ^{p- 2}\nabla v\bigr) \cdot\nabla d_{n} g{_{n}}(u-v)\,dx \biggr\vert \\& \quad = \biggl\vert \int_{\Omega\setminus D_{n}} d^{\alpha}\bigl( \vert \nabla u \vert ^{p- 2}\nabla u - \vert \nabla v \vert ^{p - 2}\nabla v\bigr) \cdot \nabla d_{n} g_{n}(u-v)\,dx \biggr\vert \\& \quad \leq \bigl\Vert d^{\alpha\frac{p-1}{p}}\bigl( \vert \nabla u \vert ^{p-1}+ \vert \nabla v \vert ^{p-1}\bigr) \bigr\Vert _{L^{q}(\Omega\setminus D_{n})} \bigl\Vert nd^{\alpha\frac{1}{p}} \bigr\Vert _{L^{p}( \Omega\setminus D_{n})} \\& \quad \leq c \biggl[ \biggl( \int_{\Omega\setminus D_{n}}d^{\alpha} \vert \nabla u \vert ^{p}\,dx \biggr) ^{\frac{1}{q}}+ \biggl( \int_{\Omega\setminus D_{n}}d^{\alpha} \vert \nabla u \vert ^{p}\,dx \biggr) ^{\frac{1}{q}} \biggr] , \end{aligned}$$

which goes to 0 as $n\rightarrow0$.

Once more, since

$$\int_{{\Omega}} \bigl\vert d^{\frac{\alpha}{p}}(u - v)_{x_{i}} \bigr\vert \,dx\leq c \biggl( \int _{{\Omega}} d^{\alpha}\bigl( \vert \nabla u \vert ^{p}+ \vert \nabla v \vert ^{p}\bigr)\,dx \biggr) ^{ \frac{1}{p}}\leq c, $$

by the Lebesgue dominated convergence theorem, we have

3.7 $$\begin{aligned}& \lim_{n \to\infty} \biggl\vert \int_{{\Omega}} \bigl({b_{i}}(u,x,t) - {b_{i}}(v,x,t)\bigr)d _{n}(x){h_{n}}(u - v) (u - v)_{x_{i}}\,dx \biggr\vert \\& \quad \leq\lim_{n \to\infty} \int_{{\Omega}} \bigl\vert {b_{i}}(u,x,t) - {b_{i}}(v,x,t) \bigr\vert \bigl\vert {h_{n}}(u - v) (u - v)_{x_{i}} \bigr\vert \,dx \\& \quad \leq c\lim_{n \to\infty} \int_{{\Omega}} \bigl\vert (u-v){h_{n}}(u - v) \bigr\vert \bigl\vert d ^{\frac{\alpha}{p}}(u - v)_{x_{i}} \bigr\vert \,dx = 0. \end{aligned}$$

Once again,

3.8 $$ \lim_{n \to\infty} \biggl\vert \int_{\Omega} \bigl({b_{i}}(u,x,t) - {b_{i}}(v,x,t)\bigr) \cdot{g}_{n}(u-v)d_{nx_{i}}(x) \,dx \biggr\vert \leq c \int_{\Omega} \vert u-v \vert \,dx. $$

Now, let $n\rightarrow\infty$ in (3.3). Then

$$\frac{d}{dt} \Vert {u - v} \Vert _{L^{1}(\Omega)} \leqslant c \Vert {u - v} \Vert _{L^{1}(\Omega)}. $$

It implies that

$$\int_{\Omega} \bigl\vert u(x,t) - v(x,t) \bigr\vert \,dx \leqslant c \int_{\Omega} \vert {u_{0}} - {v_{0}} \vert \,dx,\quad\forall t \in[0,T). $$

Theorem 1.3 is proved. □

## Another kind of weak solution

In this section, we introduce another kind of weak solution and prove another stability theorem.

### Definition 4.1

If a function $u(x,t)$ satisfies (1.10), and

4.1 $$ \iint_{{Q_{T}}} \Biggl[ u_{t}g(\varphi) + d^{\alpha} \vert \nabla u \vert ^{p- 2}\nabla u \cdot\nabla g(\varphi) +\sum _{i=1}^{N}b_{i}(u,x,t)g _{x_{i}}(\varphi) \Biggr] \,dx\,dt= 0, $$

for $\varphi\in{ C_{0}^{1} ({Q_{T}})}$, $g(s)$ is a $C^{1}$ function with $g(0)=0$, the initial value (1.7) is satisfied in the sense of (1.12), then we say $u(x,t)$ is a weak solution of Eq. (1.6) with the initial value (1.7).

Only if we choose $\varphi_{1}=g(\varphi)$, $\varphi_{2}=1$ in Definition 1.1, one can obtain the existence of the weak solutions in the sense of Definition 4.1.

### Theorem 4.2

*If* $b_{i}$ *is a Lipchitz function*,

4.2 $$\begin{aligned}& \bigl\vert b_{i}(u, x,t)-b_{i}(v,x,t) \bigr\vert \leq c g(x) \vert u-v \vert , \end{aligned}$$

4.3 $$\begin{aligned}& \int_{\Omega} g(x)d^{-1}(x)\,dx\leq c, \end{aligned}$$

*and one of the following conditions is true*:

(i) $\alpha\geq p$;
(ii) $p>\alpha>p-1$, $p>2$;

*then the stability*

4.4 $$ \int_{\Omega} \bigl\vert u(x,t) - v(x,t) \bigr\vert \,dx \leqslant \int_{\Omega} \bigl\vert u_{0}(x)- v_{0}(x) \bigr\vert \,dx,\quad\forall t \in[0,T), $$

*is true for the solutions* *u* *and* *v* *with the initial values* $u_{0}(x)$ *and* $v_{0}(x)$, *respectively*.

### Proof

By a process of limit, we may choose $\varphi= \chi_{[\tau,s]}g_{n}(d^{\beta}(u-v))$ as a test function, where *β* is a constant to be chosen later. Then

4.5 $$\begin{aligned}& \iint_{Q_{\tau s}}g_{n}\bigl((u-v)d^{\beta}\bigr) \frac{\partial(u-v)}{\partial t}\,dx\,dt \\& \quad =- \iint_{Q_{\tau s}}d^{\alpha}\bigl( \vert \nabla u \vert ^{p-2}\nabla u- \vert \nabla v \vert ^{p-2} \nabla v\bigr) \nabla\bigl[g_{n}\bigl((u-v)d^{\beta}\bigr)\bigr] \,dx\,dt \\& \quad\quad{} - \iint_{Q_{\tau s}}\bigl[b_{i}(u,x,t)-b_{i}(v,x, t)\bigr] \bigl[g_{n}\bigl((u-v)d^{\beta}\bigr) \bigr]_{x _{i}} \,dx\,dt. \end{aligned}$$

Now, let us calculate every term in (4.5). For the first term on the right hand side of (4.5),

4.6 $$\begin{aligned}& \iint_{Q_{\tau s}}d^{\alpha}\bigl( \vert \nabla u \vert ^{p-2}\nabla u- \vert \nabla v \vert ^{p-2} \nabla v\bigr) \nabla\bigl[g_{n}\bigl((u-v)d^{\beta}\bigr)\bigr] \,dx\,dt \\& \quad = \iint_{Q_{\tau s}}d^{\alpha+\beta}h_{n} \bigl((u-v)d^{\beta}\bigr) \bigl( \vert \nabla u \vert ^{p-2} \nabla u- \vert \nabla v \vert ^{p-2}\nabla v\bigr)\nabla(u-v) \,dx \,dt \\& \quad \quad{} +\beta \iint_{Q_{\tau s}}d^{\alpha+\beta-1}h_{n} \bigl((u-v)d^{\beta}\bigr) (u-v) \bigl( \vert \nabla u \vert ^{p-2}\nabla u- \vert \nabla v \vert ^{p-2}\nabla v\bigr) \nabla d \,dx\,dt. \end{aligned}$$

Clearly,

4.7 $$ \iint_{Q_{\tau s}}d^{\alpha+\beta}h_{n} \bigl((u-v)d^{\beta}\bigr) \bigl( \vert \nabla u \vert ^{p-2} \nabla u- \vert \nabla v \vert ^{p-2}\nabla v\bigr)\nabla(u-v) \,dx \,dt\geq0. $$

By the fact that $\vert \nabla d \vert =1$ is true almost everywhere, $\alpha>p-1$, we have

$$\iint_{Q_{T}}d^{\alpha-p}\,dx\,dt\leq c, $$

accordingly, using the Lebesgue dominated convergent theorem and the limit $\lim_{n \rightarrow\infty}s h_{n}(s)=0$, we have

4.8 $$\begin{aligned}& \biggl\vert \iint_{Q_{\tau s}}d^{\alpha+\beta-1}\bigl( \vert \nabla u \vert ^{p-2}\nabla u- \vert \nabla v \vert ^{p-2}\nabla v\bigr) (u-v)h_{n}\bigl((u-v)d^{\beta}\bigr)\nabla d \,dx\,dt \biggr\vert \\& \quad \leq c \biggl( \int_{\tau}^{s} \int_{\Omega}d^{\alpha}\bigl( \vert \nabla u \vert ^{p}+ \vert \nabla v \vert ^{p}\bigr)\,dx\,dt \biggr) ^{\frac{p-1}{p}} \\& \quad \quad{}\times \biggl( \int_{\tau} ^{s} \int_{\Omega}d^{\alpha}d^{p(\beta-1)} \vert \nabla d \vert ^{p} \bigl\vert h_{n}\bigl((u-v)d ^{\beta} \bigr) (u-v) \bigr\vert ^{p}\,dx\,dt \biggr) ^{\frac{1}{p}} \\ & \quad \leq c \biggl( \int_{\tau}^{s} \int_{\Omega}d^{\alpha-p} \bigl\vert h_{n} \bigl((u-v)d ^{\beta}\bigr)d^{\beta}(u-v) \bigr\vert ^{p}\,dx\,dt \biggr) ^{\frac{1}{p}}, \end{aligned}$$

which goes to zero as $n\rightarrow\infty$.

As for the second term on the right hand side of (5.5),

4.9 $$\begin{aligned}& \iint_{Q_{\tau s}}\bigl[b_{i}(u,x,t)-b_{i}(v,x,t) \bigr] \bigl[g_{n}\bigl((u-v)d^{\beta}\bigr) \bigr]_{x _{i}} \,dx\,dt \\ & \quad = \iint_{Q_{\tau s}}\bigl[b_{i}(u,x,t)-b_{i}(v,x,t) \bigr](u-v)h_{n}\bigl((u-v)d^{ \beta}\bigr)d^{\beta}_{ x_{i}} \,dx\,dt \\ & \quad \quad{} + \iint_{Q_{\tau s}}\bigl[b_{i}(u,x,t)-b_{i}(v,x,t) \bigr](u-v)_{x_{i}}d^{\beta}h _{n}\bigl((u-v)d^{\beta} \bigr)\,dx\,dt. \end{aligned}$$

Since for any given $(x,t)$, $b_{i}(s,x,t)$ is a Lipschitz function, $u,v\in L^{\infty}(Q_{T})$, we have

4.10 $$\begin{aligned} \begin{aligned}[b] & \iint_{Q_{\tau s}}\bigl[b_{i}(u,x,t)-b_{i}(v,x,t) \bigr]h_{n}\bigl((u-v)d^{\beta}\bigr) (u-v)d ^{\beta}_{x_{i}} \,dx\,dt \\ &\quad =\beta \int_{\tau}^{s} \int_{\Omega}\bigl[b_{i}(u,x,t)-b_{i}(v,x,t) \bigr]d^{-1}h _{n}\bigl((u-v)d^{\beta}\bigr) (u-v)d^{\beta} d_{ x_{i}}\,dx\,dt, \end{aligned} \end{aligned}$$

which goes to zero when $n\rightarrow0$. This is due to $[b_{i}(u,x,t)-b _{i}(v,x,t)]d^{-1}(x)\in L^{1}(Q_{T})$ by (4.2)–(4.3), using the Lebesgue dominated convergent theorem in (4.10) and using $\lim_{n \rightarrow\infty}sh_{n}(s)=0$ again.

Meanwhile, also using the dominated convergent theorem, we have

4.11 $$\begin{aligned}& \biggl\vert \iint_{Q_{\tau s}}\bigl[b_{i}(u,x,t)-b_{i}(v,x,t) \bigr](u-v)_{x_{i}} d^{\beta }h_{n}\bigl((u-v)d^{\beta} \bigr)\,dx\,dt \biggr\vert \\ & \quad \leq \biggl( \int_{\tau}^{s} \int_{\Omega}d^{(-\frac{\alpha}{p})q}\bigl[h _{n} \bigl((u-v)d^{\beta}\bigr)d^{\beta } \bigl\vert b_{i}(u,x,t)-b_{i}(v,x,t) \bigr\vert \bigr]^{q}\,dx\,dt \biggr) ^{\frac{1}{q}} \\ & \quad \quad{} \times \biggl( \int_{\tau}^{s} \int_{\Omega}d^{\alpha}\bigl( \vert \nabla u \vert ^{p}+ \vert \nabla v \vert ^{p}\bigr) \,dx\,dt \biggr) ^{\frac{1}{p}} \\ & \quad \leq c \biggl( \int_{\tau}^{s} \int_{\Omega}d^{(1-\frac{\alpha}{p})q}\bigl[h _{n} \bigl((u-v)d^{\beta}\bigr)d^{\beta} \vert u-v \vert \bigr]^{q}\,dx\,dt \biggr) ^{\frac{1}{q}}, \end{aligned}$$

which goes to zero provided that one of the conditions (i) and (ii) is true. Here $q=\frac{p}{p-1}$ as usual.

At last,

4.12 $$\begin{aligned}& \lim_{n\rightarrow\infty} \iint_{Q_{\tau s}}g_{n}\bigl((u-v)d^{\beta}\bigr) \frac{ \partial(u-v)}{\partial t}\,dx\,dt \\ & \quad = \iint_{Q_{\tau s}}\operatorname{sign}\bigl((u-v)d^{\beta}\bigr) \frac{\partial(u-v)}{ \partial t}\,dx\,dt \\ & \quad = \iint_{Q_{\tau s}}\operatorname{sign}\bigl((u-v)\bigr) \frac{\partial(u-v)}{\partial t}\,dx \,dt \\ & \quad = \int_{\Omega} \bigl\vert u(x,s)-v(x,s) \bigr\vert \,dx- \int_{\Omega} \bigl\vert u(x,\tau)-v(x,\tau ) \bigr\vert \,dx. \end{aligned}$$

By (4.6)–(4.12), we have

4.13 $$ \int_{\Omega} \bigl\vert u(x,s)-v(x,s) \bigr\vert \,dx\leq \int_{\Omega} \bigl\vert u(x,\tau)-v(x, \tau) \bigr\vert \,dx. $$

Then

$$ \int_{\Omega} \bigl\vert u(x,s)-v(x,s) \bigr\vert \,dx\leq \int_{\Omega} \bigl\vert u_{0}(x)-v _{0}(x) \bigr\vert \,dx. $$

The proof is complete. □

### Proof of Theorem 1.6

Since $\alpha>p-1$, $p>2$ and the condition (1.18) in Theorem 1.6, one can see that (4.2)–(4.3) are all right. Thus, Theorem 1.6 is true. □

## Proof of Theorem 1.7

### Proof

For a small positive constant $\lambda>0$, define

5.1 $$ \phi(x)= \textstyle\begin{cases} 1, & \text{if } x\in\Omega_{\lambda}, \\ \frac{d(x)}{\lambda}, & \text{if } x\in\Omega\setminus\Omega_{\lambda}, \end{cases} $$

where

$$\Omega_{\lambda}=\bigl\{ x\in\Omega: d(x)=\operatorname{dist}(x,\partial \Omega)> \lambda\bigr\} . $$

Then

$$\nabla\phi=\frac{1}{\lambda}\nabla d,\quad x\in\Omega\setminus \Omega_{\lambda}. $$

*u* and *v* are two weak solutions of Eq. (1.6) with the same partial homogeneous boundary value (1.20) and with the different initial values $u_{0}(x)$ and $v_{0}(x)$, respectively. According to Definition 4.1, we choose ${g_{n}}(\phi(u - v))$ as the test function. Thus

5.2 $$\begin{aligned}& \int_{\Omega}g_{n}\bigl(\phi(u - v)\bigr) \frac{\partial(u - v)}{\partial t}\,dx \\& \quad \quad {} + \int_{\Omega} d^{\alpha}\bigl( \vert \nabla u \vert ^{p - 2}\nabla u - \vert \nabla v \vert ^{p - 2}\nabla v\bigr) \cdot\phi\nabla(u - v)h _{n}\bigl(\phi(u-v)\bigr) \,dx \\& \quad \quad {} + \int_{\Omega} d^{\alpha}\bigl( \vert \nabla u \vert ^{p- 2}\nabla u - \vert \nabla v \vert ^{p - 2}\nabla v\bigr) \cdot\nabla\phi(u - v)h _{n}\bigl(\phi(u-v)\bigr) \,dx \\& \quad \quad {} + \sum_{i=1}^{N} \int_{\Omega} \bigl(b_{i}(u,x,t) - b_{i}(v,x,t) \bigr) (u - v)_{x _{i}}h_{n}\bigl(\phi(u-v)\bigr)\phi \,dx \\& \quad \quad {} +\sum_{i=1}^{N} \int_{\Omega} \bigl(b_{i}(u,x,t) - b_{i}(v,x,t) \bigr)\phi_{x _{i}} (u - v)h_{n}\bigl(\phi(u-v)\bigr) \,dx \\& \quad = 0. \end{aligned}$$

For the terms on the left hand side of (5.2),

5.3 $$\begin{aligned}& \lim_{n\rightarrow\infty}\lim_{\lambda\rightarrow0} \int_{\Omega} g_{n}\bigl(\phi(u - v) \bigr)\frac{\partial(u - v)}{\partial t}\,dx = \frac{d}{dt} \int_{\Omega} \vert u-v \vert \,dx, \end{aligned}$$

5.4 $$\begin{aligned}& \int_{\Omega} d^{\alpha}\bigl( \vert \nabla u \vert ^{p - 2}\nabla u - \vert \nabla v \vert ^{p - 2}\nabla v\bigr) \cdot\phi\nabla(u - v)h _{n}\bigl(\phi(u-v)\bigr) \,dx \geq0. \end{aligned}$$

By the fact that

5.5 $$ \bigl\vert (u - v)h_{n}\bigl(\phi(u-v)\bigr) \bigr\vert = \bigl\vert \phi(u-v)h_{n}\bigl(\phi(u-v)\bigr) \bigr\vert \frac{1}{ \phi}\leq\frac{c}{\phi}, \quad\quad \frac{ \vert \nabla\phi \vert }{\phi}\leq \frac{c}{ \lambda}, $$

using the Young inequality, we have

5.6 $$\begin{aligned}& \biggl\vert \int_{\Omega} d^{\alpha}\bigl( \vert \nabla u \vert ^{p- 2}\nabla u - \vert \nabla v \vert ^{p- 2}\nabla v\bigr) \cdot\nabla\phi(u - v)h _{n}\bigl(\phi(u-v)\bigr) \,dx \biggr\vert \\& \quad \leq \int_{\Omega\setminus\Omega_{\lambda}}d^{\alpha} \bigl( \vert \nabla u \vert ^{p - 1}+ \vert \nabla v \vert ^{p - 1}\bigr)\frac{ \vert \nabla\phi \vert }{\phi} \bigl\vert \phi(u - v) \bigr\vert h_{n}\bigl(\phi(u-v)\bigr) \,dx \\& \quad \leq c \int_{\Omega\setminus\Omega_{\lambda}}\frac{1}{\lambda}d ^{\alpha} \bigl( \vert \nabla u \vert ^{p - 1}+ \vert \nabla v \vert ^{p - 1}\bigr) \bigl\vert \phi(u - v) \bigr\vert h_{n}\bigl(\phi(u-v)\bigr) \,dx \\& \quad \leq\frac{c}{\lambda} \int_{\Omega\setminus\Omega_{\lambda}}d^{ \alpha-\frac{\alpha}{p-1}}\rho^{\frac{\alpha}{p-1}} \bigl( \vert \nabla u \vert ^{p - 1}+ \vert \nabla v \vert ^{p - 1}\bigr) \,dx \\& \quad \leq c \int_{\Omega\setminus\Omega_{\lambda}} \biggl[ d^{\alpha} \bigl( \vert \nabla u \vert ^{p}+ \vert \nabla v \vert ^{p}\bigr) + \frac{1}{ \lambda^{p}}d^{p(\alpha-\frac{\alpha}{p-1})} \biggr] \,dx, \end{aligned}$$

which goes to 0 as $\lambda\rightarrow0$, by $p-1>\alpha\geq \frac{p-1}{p-2}$, implying

$$\frac{1}{\lambda^{p}}d^{p(\alpha-\frac{\alpha}{p-1})}\leq \lambda^{[\alpha-1-\frac{\alpha}{p-1}]p}\rightarrow0. $$

Meanwhile,

5.7 $$\begin{aligned} \begin{aligned}[b] &\sum_{i=1}^{N} \biggl\vert \int_{{\Omega}} {\bigl(b_{i}(u,x,t) - b_{i}(v,x,t)\bigr)h _{n}\bigl(\phi(u - v)\bigr){{(u - v)}} \phi_{{x_{i}}}(x)\,dx} \biggr\vert \\ &\quad \leq c\sum_{i=1}^{N} \int_{{\Omega\setminus\Omega_{\lambda}}}\frac{ \vert b _{i}(u,x,t)-b_{i}(v,x,t) \vert }{\lambda}\,dx. \end{aligned} \end{aligned}$$

We use $\vert b_{i}(u,x,t)-b_{i}(v,x,t) \vert \leq a_{i}(x) \vert u-v \vert $. According to the definition of the trace, by the partial boundary value condition (1.6),

$$u(x,t)=v(x,t)=0, \quad x\in\Sigma_{1}=\Biggl\{ x\in\partial\Omega: \sum _{i=1} ^{N}a_{i}(x)\neq0\Biggr\} $$

and

$$\sum_{i=1}^{N}a_{i}(x)=0, \quad x \in \Sigma_{2}= \Biggl\{ x\in\partial\Omega : \sum _{i=1}^{N}a_{i}(x)=0 \Biggr\} , $$

we have

5.8 $$\begin{aligned} \begin{aligned}[b] &\lim_{\lambda\rightarrow0} \biggl\vert \int_{{\Omega}} {\bigl(b_{i}(u,x,t) - b _{i}(v,x,t)\bigr)h_{n}\bigl(\phi(u - v)\bigr){{(u - v)}} \phi_{{x_{i}}}(x)\,dx} \biggr\vert \\ &\quad \leq c\sum_{i=1}^{N} \int_{\partial\Omega} \bigl\vert a_{i}(x) \bigr\vert \vert u-v \vert \,d\Sigma=c \sum_{i=1}^{N} \int_{\Sigma_{1}\cup\Sigma_{2}} \bigl\vert a_{i}(x) \bigr\vert \vert u-v \vert \,d \Sigma=0.\end{aligned} \end{aligned}$$

Moreover, as in [10], we can prove that

5.9 $$ \lim_{n \to\infty} \lim_{\lambda\rightarrow0} \int_{\Omega} \bigl(b _{i}(u,x,t) - b_{i}(v,x,t) \bigr)h_{n}\bigl(\phi(u - v)\bigr) (u - v)_{x_{i}}\phi(x)\,dx = 0. $$

In detail,

5.10 $$\begin{aligned}& \lim_{\lambda\rightarrow0} \biggl\vert \int_{\Omega} \bigl(b_{i}(u,x,t) - b _{i}(v,x,t)\bigr)h_{n}\bigl(\phi(u - v)\bigr) (u - v)_{x_{i}}\phi(x)\,dx \biggr\vert \\& \quad = \biggl\vert \int_{\{x\in\Omega: \vert u - v \vert < \frac{1}{n}\}}\bigl[b_{i}(u,x,t) - b_{i}(v,x,t) \bigr]h_{n}(u - v) (u - v)_{x_{i}}\,dx \biggr\vert \\& \quad \leqslant c \int_{\{x\in\Omega: \vert u - v \vert < \frac{1}{n}\}} \biggl\vert \frac{ {b_{i}}(u,x,t) - {b_{i}}(v,x,t)}{u - v} \biggr\vert \bigl\vert (u - v)_{x_{i}} \bigr\vert \,dx \\& \quad = c \int_{\{x\in\Omega: \vert u - v \vert < \frac{1}{n}\}} \biggl\vert d^{-\frac{ \alpha}{p}}\frac{{b_{i}}(u,x,t) - {b_{i}}(v,x,t)}{u - v} \biggr\vert \bigl\vert d ^{\frac{\alpha}{p}}(u - v)_{x_{i}} \bigr\vert \,dx \\& \quad \leqslant c \biggl[ \int_{\{x\in\Omega: \vert u - v \vert < \frac{1}{n}\}} \biggl\vert d ^{-\frac{\alpha}{p}} \frac{{b_{i}}(u,x,t) - {b_{i}}(v,x,t)}{u - v} \biggr\vert ^{\frac{p}{p- 1}}\,dx \biggr] ^{\frac{p - 1}{p}} \\& \quad \quad{}\times \biggl[ \int_{\{x\in \Omega: \vert u - v \vert < \frac{1}{n}\}} \bigl\vert d^{\alpha}\nabla(u - v) \bigr\vert ^{p}\,dx \biggr] ^{\frac{1}{p}} . \end{aligned}$$

Since $\alpha< p-1$, $\vert b_{i}(u,x,t)-b_{i}(v,x,t) \vert \leq c \vert u-v \vert $,

5.11 $$ \int_{\{x\in\Omega: \vert u - v \vert < \frac{1}{n}\}} \biggl\vert d^{- \frac{\alpha }{p}}\frac{b_{i}(u,x,t) - b_{i}(v,x,t)}{u - v} \biggr\vert ^{\frac{p}{p- 1}}\,dx \leqslant c \int_{\Omega} {{d^{ - \frac{\alpha}{{p- 1}}}}\,dx} \leqslant c. $$

If $\{ x \in\Omega: \vert u - v \vert = 0\}$ is a set with 0 measure, then

5.12 $$ \lim_{n \to\infty} \int_{\{x\in\Omega: \vert u - v \vert < \frac{1}{n}\} } { \bigl\vert {d ^{\frac{\alpha}{ {p- 1}}}} \bigr\vert \,dx} = \int_{\{x\in\Omega: \vert u - v \vert =0\}} { \bigl\vert {d^{\frac{ \alpha}{{p- 1}}}} \bigr\vert \,dx} = 0. $$

If the set $\{ x \in\Omega: \vert u - v \vert = 0\}$ has a positive measure, then

5.13 $$ \lim_{n\to\infty} \int_{\{x\in\Omega: \vert u - v \vert < \frac{1}{n}\}} {d^{\alpha}} \bigl\vert \nabla(u - v) \bigr\vert ^{p}\,dx = \int_{\{x\in\Omega: \vert u - v \vert = 0\}} {d^{\alpha}} \bigl\vert \nabla(u - v) \bigr\vert ^{p}\,dx = 0. $$

Therefore, in both cases, (5.10) goes to 0 as $\eta\rightarrow0$.

Now, after letting $\lambda\rightarrow0$, let $n\rightarrow\infty $ in (5.2). Then, by (5.3), (5.4), (5.6), (5.8), and (5.9), we have

$$\frac{d}{dt} \int_{\Omega} \vert u-v \vert \,dx \leqslant c \int_{\Omega} \vert u-v \vert \,dx, $$

by the Gronwall inequality, we have

$$\int_{\Omega} \bigl\vert {u(x,t) - v(x,t)} \bigr\vert \,dx \leqslant c \int_{\Omega} \bigl\vert {{u_{0}}(x) - {v_{0}}} (x) \bigr\vert \,dx,\quad \forall t \in[0,T), $$

Theorem 1.7 is proved. □

## The partial boundary condition

Let us simply review Fichera–Oleǐnik theory. For a linear degenerate elliptic equation,

6.1 $$ \sum_{r,s=1}^{N+1}a^{rs}(x) \frac{\partial^{2} u}{\partial x_{r}\partial x_{s}}+\sum_{r=1}^{N+1} b_{r}(x)\frac{\partial u}{\partial x_{r}}+c(x)u=f(x), \quad x\in \widetilde{\Omega}\subset \mathbb{R}^{N+1}, $$

the symmetric matrix $( a^{rs}(x) ) $ has nonnegative characteristic value, to study its well-posedness problem, one only needs to give a partial boundary condition. In detail, let $\{n_{s}\}$ be the unit inner normal vector of *∂*Ω̃ and denote

$$\begin{aligned}& \Sigma_{2}=\bigl\{ x\in\partial\widetilde{\Omega}: a^{rs}n_{r}n_{s}=0, \bigl(b _{r}-a^{rs}_{x_{s}} \bigr)n_{r}< 0\bigr\} , \\& \Sigma_{3} =\bigl\{ x\in\partial\widetilde{\Omega}: a^{rs}n_{s}n_{r}>0\bigr\} . \end{aligned}$$

Then, to ensure the well-posedness of Eq. (1.7), Fichera–Oleǐnik theory tells us that the suitable boundary condition is

6.2 $$ u| _{\Sigma_{2}\cup\Sigma_{3}}=g(x). $$

In particular, if the matrix $(a^{rs})$ is positive definite, (6.2) is just the usual Dirichlet boundary condition. Considering the classical parabolic equation

6.3 $$ u_{t}=\sum_{i,j=1}^{N}a^{ij}(x) \frac{\partial^{2} u}{\partial x_{i} \partial x_{j}}+\sum_{i=1}^{N} b_{i}(x,t)\frac{\partial u}{\partial x _{i}}+c(x,t)u=f(x,t), $$

with the matrix $(a^{ij})$ is positive definite, besides the initial condition

6.4 $$ u(x,0)=u_{0}(x), \quad x\in\Omega, $$

only a parabolic boundary value condition

6.5 $$ u(x,t)=g(x,t),\quad (x,t)\in\partial\Omega\times[0,T), $$

is imposed. However, for Eq. (1.6) considered in this paper, since the equations are strongly nonlinear and degenerate, including the extremely case of $a\equiv0$, Fichera–Oleǐnik theory is invalid, the corresponding problem becomes more complicated. To show that the partial boundary value condition imposed on the main equation (1.6) is reasonable, we can come back to the linear case. In other words, let us suppose that $p=2$ and

6.6 $$ b_{i}(u,x,t)=a_{i}(x)u. $$

Then Eq. (1.6) has the form

6.7 $$ {u_{t}} = \operatorname{div} \bigl(d^{\alpha}\nabla u\bigr) + \sum_{i=1}^{N}a _{i}(x) \frac{\partial u}{\partial{x_{i}}}+u\operatorname{div} \overrightarrow{a},\quad(x,t) \in{Q_{T}}, $$

where $\overrightarrow{a}=\{a_{i}\}$. According to Fichera–Oleǐnik theory, the optional boundary value condition is

6.8 $$ u(x,t)=0, \quad (x,t)\in\Sigma\times[0,T), $$

with

6.9 $$ \Sigma=\bigl\{ x\in\partial\Omega: a_{i}(x)n_{i}(x)< 0\bigr\} , $$

where $\vec{n}=\{n_{i}\}$ is the inner normal vector of Ω.

Now, by reviewing the partial boundary value condition (1.24)

$$\Sigma_{p}=\Biggl\{ x\in\partial\Omega: \sum _{i=1}^{N}a_{i}(x)\neq0\Biggr\} , $$

we have found

6.10 $$ \Sigma\subseteq\Sigma_{p}. $$

Though the condition (1.24) may be not the optimal, it is reasonable.

## Conclusion

Besides the diffusion coefficient $d^{\alpha}$ being degenerate on the boundary, Eq. (1.6) has a convection term $\sum_{i=1}^{N}\frac{\partial b_{i}(u,x,t)}{\partial x_{i}}$, which depends on the spatial variable *x*. Such a characteristic can bring about essential changes on the boundary value condition. A reasonable partial boundary value condition is proposed for the first time, the stability of the weak solutions based on this partial boundary value condition is established. One can see that, if the convection term is independent of the spatial variable *x*, putting up a reasonable partial boundary condition becomes more difficult. We hope we can solve this problem in our follow-up work.
